# Immunohistochemical staining of ERG and SOX9 as potential biomarkers of docetaxel response in patients with metastatic castration-resistant prostate cancer

**DOI:** 10.18632/oncotarget.13407

**Published:** 2016-11-16

**Authors:** Wan Song, Ghee Young Kwon, Jeong Hoon Kim, Joung Eun Lim, Hwang Gyun Jeon, Seong Il Seo, Seong Soo Jeon, Han Yong Choi, Byong Chang Jeong, Hyun Moo Lee

**Affiliations:** ^1^ Department of Urology, Samsung Medical Center, Sungkyunkwan University School of Medicine, Seoul, Korea; ^2^ Department of Pathology, Samsung Medical Center, Sungkyunkwan University School of Medicine, Seoul, Korea; ^3^ Department of Molecular Biology, Samsung Advanced Institute for Health Science and Technology, Sungkyunkwan University School of Medicine, Seoul, Korea; ^4^ Department of Urology, Samsung Biomedical Research Institute, Samsung Medical Center, Seoul, Korea

**Keywords:** biomarker, docetaxel, ERG, prostate cancer, SOX9

## Abstract

We aimed to evaluate ERG and SOX9 as potential biomarkers of docetaxel response in metastatic castration-resistant prostate cancer (mCRPC) patients. Seventy-one mCRPC patients were evaluated. Tissue microarrays were constructed and immunohistochemistry was performed. Treatment response was assessed by prostate specific antigen (PSA) response rate, PSA progression-free survival (PSA-PFS), clinical/radiologic PFS (C/R-PFS) and overall survival (OS). ERG and SOX9 were found in 13 (18.3%) and 62 (87.3%) patients, respectively. ERG-positive had lower PSA response rates than negative (15.4% vs 62.1%, *p* = 0.004), and SOX9 showed a same trend (46.8% vs 100.0%, *p* = 0.003). ERG positivity correlated with a lower PSA-PFS (3.2 mos vs 7.4 mos, *p* < 0.001), C/R-PFS (3.8 mos vs 9.0 mos, *p* < 0.001) and OS (10.8 mos vs 21.4 mos, *p* < 0.001). SOX9 positivity also showed a lower PSA-PFS, C/R-PFS and OS (*p* =0.006, *p* =0.012 and *p* =0.023, respectively). On multivariate analysis, ERG positivity was a significant risk factor for a lower PSA-PFS, C/R-PFS and OS (*p* < 0.001, *p* < 0.001 and *p* =0.001, respectively). SOX9 expression was also a risk factor for a lower PSA-PFS, C/R-PFS and OS (*p* = 0.018, *p* = 0.025 and *p* =0.047, respectively). These findings indicate that ERG and SOX9 is potential biomarkers for prediction to docetaxel treatment in mCRPC patients.

## INTRODUCTION

Treatment of metastatic castration-resistant prostate cancer (mCRPC) is a major clinical challenge. Patients with mCRPC have a poor prognosis with an expected survival time less than 2 years [1]. Docetaxel-based chemotherapy is recommended as first-line standard of care for mCRPC [2] based on the results of two phase III studies (Southwest Oncology Group [SWOG] 9916 and Taxotere [Tax] 327) that demonstrated a significant survival benefit [3, 4]. However, most of the patients eventually developed treatment resistance and experienced treatment-related toxicity [5, 6], thereby underscoring the need for a biomarker for prediction to docetaxel treatment.

Recently, several molecular studies have elucidated the mechanisms for docetaxel resistance and have broadened our understanding of mCRPC [7–9]. E26 transformation-specific (ETS)-related gene (ERG) expression was increased 30 to 80 times above normal levels in approximately 50% of prostate cancer [10, 11]. An *in vitro* study showed that overexpressed ERG binds to microtubules and alters their dynamics. This also inhibits drug-target engagement, thus leading to docetaxel resistance [10, 12]. In addition, two studies examined the function of ERG and identified SRY-related HMG box (SOX) 9 as an important downstream effector of ERG [13, 14]. Therefore, expression of ERG and SOX9 in mCRPC patients might influence on the treatment outcomes.

In this study, we constructed tissue microarrays (TMAs) using prostate biopsy samples and carried out immunohistochemistry (IHC) analyses to evaluate the clinical utility of ERG and SOX9 as potential biomarkers of docetaxel response in mCRPC patients.

## RESULTS

The baseline characteristics of 71 patientswith mCRPC who underwent docetaxel treatment are presented in Table 1. At the time of diagnosis, the mean age and prostate specific antigen (PSA) were 64.9 (7.5, 49.0-88.0) years and 775.7 (1597.0, 4.6-7539.3) ng/ml, respectively. The mean duration of androgen deprivation therapy (ADT) use prior to docetaxel treatment was 28.6 (20.6, 3.3-94.3) months, and the mean PSA nadir after ADT was 5.1 (11.8, 0.01-65.66) ng/ml. Forty-seven (66.2%) patients had a high metastatic burden at the time of docetaxel treatment. During a mean follow-up period of 21.6 (14.7, 3.2-86.8) months post-docetaxel treatment, all patients developed both PSA and C/R progression, 54 (76.1%) of whom died. When patients were divided depending on ERG expression, baseline characteristics of mCRPC patients were not significantly different except initial PSA.

**Table 1:** Baseline characteristics of mCRPC patients

Variables	Total	Immunohistochemistry		*P*
		ERG(+)	ERG(−)	
No. of patients	71 (100)	13 (18.3)	58 (81.7)	
Age at diagnosis, years	64.9 ± 7.5 [64.0, 49.0-88.0]	63.5 ± 10.6 [62.0, 49.0-88.0]	65.2 ± 6.6 [65.5, 52.0-78.0]	0.450
Gleason score at diagnosis				0.346
7	9 (12.7)	1 (7.7)	8 (13.8)	
8	19 (26.8)	3 (23.1)	16 (27.6)	
9	32 (45.1)	6 (46.1)	26 (44.8)	
10	11 (15.5)	3 (23.1)	8 (13.8)	
Initial PSA, ng/ml	775.7 ± 1597.0 [166.6, 4.6-7539.3]	170.9 ± 174.8 [134.2, 8.7-551.0]	916.1 ± 1743.6 [225.9, 4.6-7539.3]	0.003
PSA nadir after ADT	5.1 ± 11.8 [1.0, 0.01-65.66]	6.5 ± 8.4 [2.0, 0.07-19.52]	4.7 ± 12.5 [0.9, 0.01-65.66]	0.624
Metastatic status before docetaxel treatment				0.798
Low volume	24 (33.8)	4 (30.8)	20 (34.5)	
High volume	47 (66.2)	9 (69.2)	38 (65.5)	
Type of local treatment				0.358
None	62 (87.3)	12 (92.3)	50 (86.2)	
Prostatectomy	3 (4.2)	1 (7.7)	2 (3.5)	
HIFU	6 (8.5)	0	6 (10.3)	
ADT duration prior to docetaxel treatment	28.6 ± 20.6 [22.9, 3.3-94.3]	22.5 ± 18.3 [17.2, 3.3-66.7]	30.0 ± 20.9 [23.7, 6.3-94.3]	0.240
No. of docetaxel regimens	6.9 ± 4.0 [6.0, 3.0-17.0]	7.3 ± 3.3 [8.0, 3.0-12.0]	6.8 ± 4.1 [5.0, 3.0-17.0]	0.707
Follow-up, months				
From initial diagnosis to docetaxel treatment	31.3 ± 21.6 [25.7, 2.1-94.0]	20.5 ± 15.0 [18.3, 2.1-41.0]	33.8 ± 22.2 [26.4, 3.4-94.0]	0.045
From docetaxel treatment to death or last visit	21.6 ± 14.7 [17.6, 3.2-86.8]	12.5 ± 7.8 [10.8, 3.2-26.2]	23.6 ± 15.1 [19.6, 4.0-86.8]	0.013
Overall	52.9 ± 27.2 [5.3-126.4]	33.0 ± 19.8 [31.1, 5.3-63.1]	57.4 ± 26.8 [51.7, 21.1-126.4]	0.003
Type of post-chemotherapy treatment				0.423
None	51 (71.9)	9 (69.2)	42 (72.5)	
Abiraterone only	4 (5.6)	2 (15.4)	2 (3.4)	
Cabazitaxel only	3 (4.2)	1 (7.7)	2 (3.4)	
Enzalutamide only	7 (9.9)	1 (7.7)	6 (10.4)	
Abiraterone/Cabazitaxel	1 (1.4)	0	1 (1.7)	
Abiraterone/Enzalutamide	4 (5.6)	0	4 (6.9)	
Abiraterone/Cabazitaxel/Enzalutamide	1 (1.4)	0	1 (1.7)	

Data are presented as means ± SD [median, range] or number (%).

SD, standard deviation; CRPC, castration-resistant prostate cancer; PSA, prostate specific antigen; ADT, androgen deprivation therapy; HIFU, high-intensity focused ultrasound.

**Figure 1:**
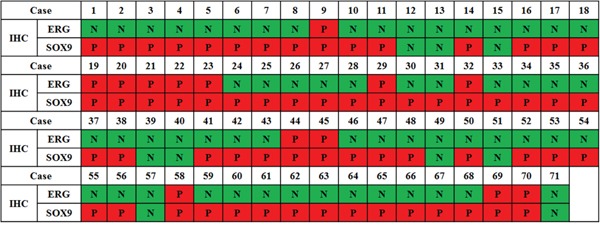
Correlation between IHC-measured ERG and SOX9 expression in 71 mCRPC patients (P, positive; N, negative)

Of the total 71 patients, ERG was positive in 13 (18.3%) patients and 62 (87.3%) patients were SOX9-positive. All patients with positive ERG expression as detected via IHC also showed SOX9 positivity. However, in patients negative for ERG expression, 49 (84.5%) were SOX9-positive and 9 (15.5%) patients were negative. The correlation of IHC results with ERG and SOX9 expression is depicted in Figure 1.

**Figure 2:**
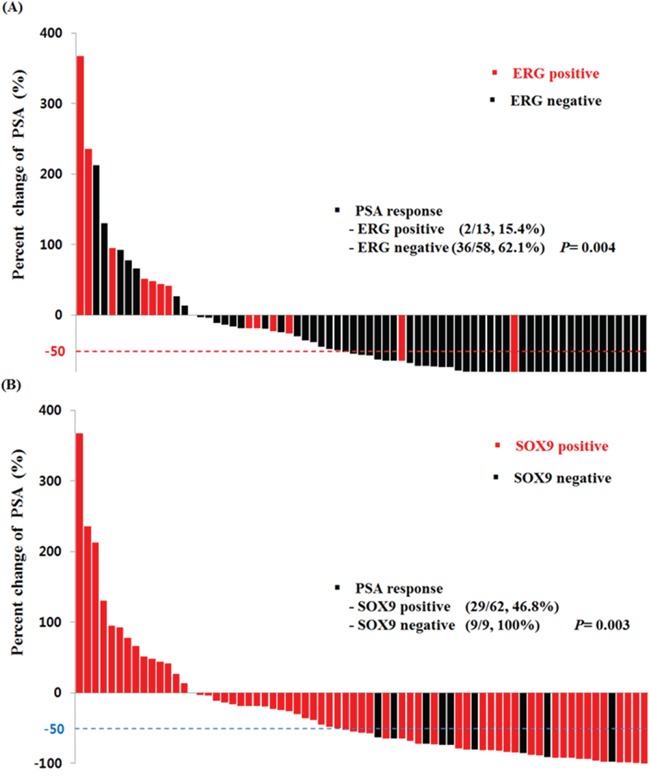
Waterfall plot of PSA levels in response to docetaxel treatment according to **A.** ERG and **B.** SOX9 expression.

**Figure 3:**
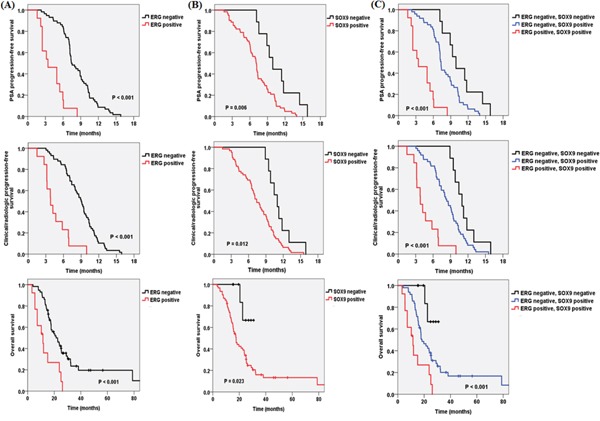
Kaplan-Meier analysis depicting PSA progression-free survival, clinical/radiologic progression-free survival and overall survival according to **A.** ERG, **B.** SOX9 and **C.** ERG and SOX9 expression.

Figure 2 shows the PSA response rate according to ERG and SOX9 IHC results. ERG-positive patients had a lower PSA response rate than negative patients (15.4% vs 62.1%, *p* = 0.004). SOX9 also presented a same trend (46.8% vs 100.0%, *p* = 0.003). The PSA-PFS, C/R-PFS and OS values estimated using the Kaplan-Meier method and the results of the log-rank test are presented in Figure 3. There were significant differences in the PSA-PFS, C/R-PFS and OS according to ERG expression (Figure 3A) (all *p* < 0.001, respectively). The median PSA-PFS was 3.2 months in ERG-positive patients and 7.4 months in negative patients. The median C/R-PFS and OS were 3.8 months and 10.8 months in ERG-positive patients, and 9.0 months and 21.4 months in ERG-negative patients, respectively. In addition, a positive SOX9 result was also correlated with a lower PSA-PFS, C/R-PFS and OS than a negative SOX9 result (Figure 3B). The median PSA-PFS and C/R-PFS were 7.1 months and 7.4 months in SOX9-positive patients, and 9.3 months and 11.0 months in SOX9-negative patients, respectively. The median OS was 19.7 months in SOX9-positive patients but not reached to median in SOX9 negative patients. When we analyzed the patients in 3 subgroups according to the combined effects of ERG and SOX9, the presence of both ERG and SOX9 positivity was significantly associated with a lower PSA-PFS, C/R-PFS and OS (Figure 3C) (all *p* < 0.001, respectively).

Table 2 shows the Cox proportional hazard regression analysis for the PSA-PFS, C/R-PFS and OS in all 71 mCRPC patients. On multivariate analysis, ERG positivity was significantly associated with a lower PSA-PFS (*p* < 0.001, hazard ratio (HR): 6.00, 95% confidence interval (CI): 2.96-12.16), C/R-PFS (*p* < 0.001, HR: 5.50, 95% CI: 2.68-11.29) and OS (*p* = 0.001, HR: 3.31, 95% CI: 1.66-6.64). In addition, SOX9 was a significant risk factor for a decreased PSA-PFS (*p* = 0.018, HR: 2.75, 95% CI: 1.19-6.32), C/R-PFS (*p* = 0.025, HR: 2.44, 95% CI: 1.12-5.30) and OS (*p* = 0.047, HR: 4.30, 95% CI: 1.02-18.16). High metastatic volume was significantly associated with a lower C/R-PFS (*p* = 0.003, HR: 2.31, 95% CI: 1.32-4.04) and OS (*p* = 0.003, HR: 2.88, 95% CI: 1.44-5.76), but not the PSA-PFS.

**Table 2:** Multivariate Cox proportional hazard regression analyses in the prediction of PSA-progression free survival (PFS), C/R-PFS and overall survival (OS) in mCRPC patients

Variables	PSA-PFS			C/R-PFS			OS		
	HR	95% CI	*p*	HR	95% CI	*p*	HR	95% CI	*p*
Age									
≤ 65.0	Ref			Ref			Ref		
> 65.0	0.76	0.46-1.28	0.306	0.91	0.54-1.53	0.715	0.79	0.45-1.39	0.420
PSA nadir after ADT									
≤ 1.0	Ref			Ref			Ref		
> 1.0	1.13	0.68-1.88	0.635	0.97	0.59-1.61	0.920	0.98	0.55-1.72	0.934
Gleason score									
7	Ref			Ref			Ref		
8-10	0.56	0.27-1.17	0.124	0.63	0.30-1.32	0.221	1.08	0.46-2.53	0.863
Metastatic volume									
Low	Ref			Ref			Ref		
High	1.57	0.89-2.78	0.122	2.31	1.32-4.04	0.003	2.88	1.44-5.76	0.003
ERG									
Negative	Ref			Ref			Ref		
Positive	6.00	2.96-12.16	< 0.001	5.50	2.68-11.29	< 0.001	3.31	1.66-6.64	0.001
SOX9									
Negative	Ref			Ref			Ref		
Positive	2.75	1.19-6.32	0.018	2.44	1.12-5.30	0.025	4.30	1.02-18.16	0.047

PSA-PFS, prostate specific antigen progression-free survival; C/R-PFS, clinical/radiologic progression-free survival; OS, overall survival; HR, hazard ratio; CI, confidence interval; ADT, androgen deprivation therapy.

## DISCUSSION

In this study, 18.3% and 87.3% of patients with mCRPC showed positive ERG and SOX9 expression per IHC analysis, respectively. When examining their associations with clinical outcomes, ERG and SOX9 were significant risk factors for lower PSA-PFS, C/R-PFA and OS after docetaxel treatment. In addition, their effects on docetaxel response were even more exaggerated when analyzed in the 3 subgroups. These results suggest that ERG and SOX9 is potential biomarkers for prediction to docetaxel treatment in mCRPC patients. To the best of our knowledge, our study is the first study to analyze the correlation between ERG and SOX9 as measured by IHC and docetaxel response in mCRPC patients.

Recent studies showed that ERG overexpression occurs in at least 50% of prostate cancer cases as a result of gene fusion, with a *TMPRESS2*-ERG rearrangement being the most common form [10, 15, 16]. In a study by Galletti *et al* [10], eleven (32.4%) patients were positive for the *TMPRESS2*-ERG fusion, and PSA response to docetaxel was associated with circulation tumor cell (CTC) ERG expression in 34 mCRPC patients (positive; 45% vs negative; 79%, p =0.056). In addition, Reig *et al* [15] reported that 8 (16%) of 50 docetaxel-treated mCRPC patients had CTCs positive for the *TMPRESS2*-ERG fusion. This finding was also significantly associated with a lower PSA response, PSA-PFS, C/R-PFS and OS. Furthermore, in the 25 docetaxel-treated mCRPC patients who had tissue samples available, *TMPRESS2*-ERG was found to be positive in 14 (56%) patients and associated with lower PSA-PFS, but not C/R-PFS.

In comparison to previous studies [10, 15], our study focused on detecting ERG expression rather than the *TMPRESS2*-ERG rearrangement. Park *et al* [17] found IHC detection of ERG to have high sensitivity, specificity and accuracy in the assessment of *TMPRESS2*-ERG fusion status, and another study found that ERG IHC expression was congruent with the *TMPRESS2*-ERG fusion [18]. These results were further validated by a larger cohort study that the ERG IHC results could be used as a simple and accurate surrogate for *TMPRESS2*-ERG fusion status detection [19]. Previous studies, however, have highlighted the different clinical implications that exist for genetic alterations in CTCs vs. tissue [15], thus it has not yet been clearly determined whether tissue or blood would provide the most clinically useful ERG IHC results.

In addition, our study showed that expression of SOX9, a known downstream effector of ERG, was also correlated with a lower PSA-PFS, C/R-PFA and OS after docetaxel treatment in mCRPC patients. During fetal growth, it is well known that SOX9 is essential for prostate development, therefore a prostate-specific SOX9 knockout model results in profoundly defective prostate morphogenesis [20]. In the adult prostate, SOX9 plays an essential role in preserving the luminal epithelium [21]. However, recent studies examining the role of SOX9 in prostate cancer showed that overexpression was associated with a higher Gleason score [22], cancer progression and invasion [23]. In xenograft models of prostate cancer, increased expression of SOX9 causes cancer growth, invasion and angiogenesis, while silencing of SOX9 dramatically decreases tumor growth [14, 23].

Wang H. *et al* [21] reported that SOX9 expression was further increased in patients with mCRPC. In our study, all ERG-positive patients were also SOX9 positive and, interestingly, 49 of 58 (84.5%) patients were negative for ERG but positive for SOX9. A possible explanation for this is the regulation of SOX9 by other pathways, including the Wnt/beta-catenin or MAP kinase pathways [13, 21]. In addition, *in vitro* studies, SOX9 expression is suppressed by androgens in ERG-negative prostate cancer cells, therefore ADT may actually induce SOX9 expression in ERG-negative patients [14, 22]. Remarkably, 9 patients with both ERG and SOX9 negativity showed more favorable clinical outcomes after docetaxel treatment when compared to the other subgroups. Therefore, SOX9 expression could be used as biomarkers of the activation of other pathways known to provoke prostate cancer progression and of docetaxel response together with the results of ERG IHC [14].

Despite the clinical implications, our study had several limitations that need to be considered for interpretation. Foremost, this study was retrospective in design, conducted at a single institution and included a relatively small patient population, thus raising concern for selection bias. However, this is the largest study to analyze the correlation between IHC-measured ERG and SOX9 and docetaxel response, as evaluated by the PSA response, PSA-PFS, C/R-PFA and OS. Second, when interpreting IHC results, we only considered intensity and did not apply other interpretation methods. Since there are no objective guidelines for interpretation, there may be a discrepancy in the results. Finally, other important prognostic factors, such as alkaline phosphatase and lactic dehydrogenase, were not considered due to lack of available information.

In conclusion, our result indicated that IHC-detected ERG and SOX9 expression is significantly associated with lower PSA-PFS, C/R-PFS and OS in patients with mCRPC treated with docetaxel. Therefore, they could be used as potential biomarkers for prediction to docetaxel treatment in mCRPC patients. Further large, prospective clinical trials are necessary to confirm our results.

## MATERIALS AND METHODS

### Study population and data collection

A retrospective study was conducted on 78 patients who were diagnosed with mCRPC and treated with docetaxel (75 mg/m^2^ intravenously, every 3 weeks) between 2001 and 2013. We excluded 7 patients who had tissue samples collected after docetaxel treatment. The tissues of the remaining 71 patients were obtained from prostate biopsy specimens at the time of diagnosis. Clinical information including follow-up data after docetaxel treatment was retrieved from the patients' medical charts. In regards to metastatic status, high volume disease was defined as the existence of visceral metastases or having more than 4 bone lesions, one of which was present beyond the vertebral bodies or pelvis [24]. To assess the therapeutic response, the serum PSA was measured after each treatment and a computed tomography (CT) and/or bone scan was performed after every third treatment. This study was carried out with the approval of the institutional review board committee at our institution.

### Response evaluation

After docetaxel treatment, we evaluated the PSA response rate, PSA progression-free survival (PSA-PFS), clinical/radiologic PFS (C/R-PFS) and the overall survival (OS). Treatment response to docetaxel was assessed based on the recommendations of the Prostate Cancer Working Group 2 (PCWG2) [25]. The PSA response was measured using the percent change of the PSA level from baseline to 12 weeks post-treatment, and a therapeutic response was defined as more than a 50% decline from baseline maintained over at least 4 weeks. The PSA-PFS, C/R-PFS and OS were estimated based on the time between the initial treatment and PSA progression, C/R progression and death or last visit. PSA progression was defined as an increase in the PSA more than 25% (at least 2 ng/ml) from the nadir that persisted for at least 3 weeks. Clinical progression was defined as a new onset or aggravation of cancer-related symptoms or a higher analgesic requirement [15]. Radiological progression was defined as two or more new lesions confirmed on a bone scan or evidence of progression on a CT scan, as per the recommendations of the Response Evaluation Criteria in Solid Tumor version 1.1 (RECIST v1.1) [15, 26].

### TMA construction

Typical formalin-fixed paraffin-embedded (FFPE) tissue blocks were obtained from hematoxylin-eosin stained slides for TMA preparation. Two, 2-mm tissue cores were taken from the donor blocks with the use of a manual tissue microarrayer (ISU ABXIS, Seoul, Republic of Korea) and placed into a recipient TMA block.

### IHC detection of ERG and SOX9 and interpretation

Paraffin-embedded TMA sections 4 μm thick were mounted on slides to determine ERG and SOX9 expression levels. The IHC for ERG was performed on a BenchMark XT automated stainer (Roche/Ventana Medical Systems, Tucson, Arizona, USA) using an ERG rabbit monoclonal antibody (EPR3864, Epitomics, Burlingame, California, USA; dilution 1:100) according to the manufacturer's instructions. Vascular endothelial cells and normal horse serum replaced by primary antibodies were used as positive and negative controls, respectively. IHC of SOX9 was performed as follows: First, the endogenous peroxidase activity was blocked by incubating each section in 0.3% hydrogen peroxide. Antigens were then retrieved by heating the sections in Target Retrieval Solution (pH 9.0) (DAKO, Denmark) at 95°C for 20 minutes. The sections were incubated overnight at 4°C with primary mouse monoclonal antibodies against SOX9 (Abnova, Taipei, Taiwan), followed by a 1-hour incubation at room temperature (RT) with ChemMate^TM^ DAKO EnVision ^TM^/HRP (DAKO, Denmark) and an additional hour at RT with DakoCytomation TechMate™ (DAKO, Denmark).

**Figure 4:**
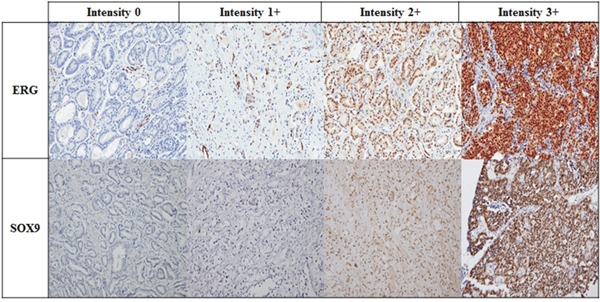
Representative images of ERG and SOX9 detection by IHC in mCRPC patients according to intensity (Magnification x 200)

The IHC results were interpreted by an experienced genitourinary pathologist (G.Y. K). The intensities of ERG and SOX9 expression in tumor cells were scored using a four-tiered grading system, which was as follows: negative (0; no staining), weak (1+; visible only at high magnification), moderate (2+; visible at low magnification) or strong (3+; prominent at low magnification) (Figure 4). A sample with more than 5% of total stained area scoring 2+ or 3+ in intensity was considered to be positive [27].

### Statistical analysis

Continuous and categorical variables were described as means (standard deviation, range) and absolute values (percentage). An independent *t*-test and Fisher's exact test or linear by linear association were used to compare clinical characteristics. Individual PSA responses were depicted by a waterfall plot and compared using the Fisher's exact test. Kaplan-Meier survival curves were constructed to illustrate the PSA-PFS, C/R-PFS and OS according to the expression patterns of ERG and SOX9, and a log rank test was employed to compare patient subgroups. Univariate and multivariate Cox proportional hazard models were utilized to estimate associations between the PSA-PFS, C/R-PFS, OS and risk factors of interest. All statistical analyses were performed with IBM SPSS version 20.0 (IBM Corp. Armonk, NY, USA). Two-tailed *p* values <0.05 were considered statistically significant.
